# A transfer learning-based multimodal neural network combining metadata and multiple medical images for glaucoma type diagnosis

**DOI:** 10.1038/s41598-022-27045-6

**Published:** 2023-07-26

**Authors:** Yi Li, Yujie Han, Zihan Li, Yi Zhong, Zhifen Guo

**Affiliations:** 1 College of Information Science and Engineering, Northeastern University, Shenyang, Liaoning China; 2 College of Software, Northeastern University, Shenyang, Liaoning China; 3 College of Metallurgy, Northeastern University, Shenyang, Liaoning China

**Keywords:** Medical research, Engineering

## Abstract

Glaucoma is an acquired optic neuropathy, which can lead to irreversible vision loss. Deep learning(DL), especially convolutional neural networks(CNN), has achieved considerable success in the field of medical image recognition due to the availability of large-scale annotated datasets and CNNs. However, obtaining fully annotated datasets like ImageNet in the medical field is still a challenge. Meanwhile, single-modal approaches remain both unreliable and inaccurate due to the diversity of glaucoma disease types and the complexity of symptoms. In this paper, a new multimodal dataset for glaucoma is constructed and a new multimodal neural network for glaucoma diagnosis and classification (GMNNnet) is proposed aiming to address both of these issues. Specifically, the dataset includes the five most important types of glaucoma labels, electronic medical records and four kinds of high-resolution medical images. The structure of GMNNnet consists of three branches. Branch 1 consisting of convolutional, cyclic and transposition layers processes patient metadata, branch 2 uses Unet to extract features from glaucoma segmentation based on domain knowledge, and branch 3 uses ResFormer to directly process glaucoma medical images.Branch one and branch two are mixed together and then processed by the Catboost classifier. We introduce a gradient-weighted class activation mapping (Grad-GAM) method to increase the interpretability of the model and a transfer learning method for the case of insufficient training data,i.e.,fine-tuning CNN models pre-trained from natural image dataset to medical image tasks. The results show that GMNNnet can better present the high-dimensional information of glaucoma and achieves excellent performance under multimodal data.

## Introduction

Glaucoma is an acquired optic neuropathy characterized high intraocular pressure(IOP), optic disc atrophy and depression, visual field defects and vision loss. It is clinically classified into three main categories: primary, secondary and congenital. According to the World Health Organization, 3.5% of people over the age of 45 now suffer from glaucoma, and the estimated number of glaucoma patients worldwide will reach 111.2 million in 2040^1^. Once diagnosed, treatment decisions depend on the rate of progression, so preventing or slowing further irreversible vision loss is extremely important for a large number of glaucoma patients. At the same time, the socioeconomic cost of glaucoma increases fourfold in the early stages compared to the end stage, and timely diagnosis and intervention can save health care resources and avoid a significant disease burden^2,3^.

With the help of medical images, deep learning has been successfully applied on a variety of medical fields. For data-driven deep learning, accurately labeled and large number of datasets will be more conducive to training accuracy beyond that of clinical experts. Although much of the data has been made publicly available for researchers to use, there are still three problems that limit in-depth research in intelligent diagnosis of glaucoma. First, the datasets are mostly fundus photographs, which are not rich enough in variety. Second, the data labels of the classification task are mostly dichotomous, which cannot meet the needs of clinical disease type diagnosis of glaucoma. The segmentation task requires manual annotation of optic disc and optic cup regions or annotation of retinal vessels, which is not only an extremely laborious task but also prone to human errors in annotation even in the case of clinical experts. Third, the number of medical images in the public dataset is insufficient to train CNNs from scratch. Different from previous glaucoma image datasets, we construct a dataset consisting of electronic medical records (text), and four medical images: fundus photographs, optical coherence tomography (OCT), ultrasound biomicroscopy(UBM), and retinal nerve fiber layer (RNFL) thickness. The dataset labels are separated into classification labels and segmentation labels. The classification labels are composed of normal and the four most important glaucoma pathotypes, and the segmentation labels annotate the optic disc and optic cup regions, retinal vessels and eye corners.

Both classification CNNs that use a binary dataset to directly output whether it is glaucoma and use a dataset labeled with segmented regions to output segmented features have received attention from many researchers and achieved great success. Although the accuracy of these methods is high, they are powerless in the diagnosis of glaucoma disease type. In this paper, we propose a multimodal neural network for glaucoma which fuses metadata and medical images using constructed multimodal glaucoma data, which provides a deeper diagnosis into glaucoma pathotypes based on categorical labels and extracts glaucoma features using segmentation annotation, details of network are described in the Methods section. In order to solve the problem of insufficient data, which is common in medical images, we introduce transfer learning methods, i.e.fine-tuning CNN models pre-trained from natural image dataset to medical image tasks, and conduct comparison experiments with models trained from scratch. To avoid the black-box feature of deep learning, we added interpretability to the model using the Grad-CAM method and conducted comparative experiments with several CAM methods.

The main contributions of the article are summarized as follows:

- A multimodal dataset(GM367) for multiclass glaucoma diagnosis is constructed which contains metadata and medical images. Note that this is the first dataset which contains labels for the five most important glaucoma categories.
- A multimodal neural network (GMNNnet) is proposed, which consists of three branches that process patient metadata, features extracted from images, and global and local details of medical images captured by deep learning model.
- In addition to data expansion, transfer learning are introduced to overcome the problems of insufficient medical image data.
- We applied the Grad-CAM method to construct interpretable visual modules and compared it with other saliency/CAM methods.

## Related works

### Glaucoma public datasets

The acquisition of large-scale, high-quality, and diverse glaucoma datasets has become one challenge.For data-driven learning, large-scale well-annotated datasets with representative data distribution characteristics are critical for learning more accurate or generalizable models^4^.We summarize the details of the glaucoma public datasets in the Table 1, including data type, quantity, data pixels and generation distribution.

### Singlemodal methods

At present, most applications of artificial intelligence in glaucoma use single modal data to deal with specific tasks. Among them, the retinal optic nerve head cup-to-disc ratio (CDR) is considered an important indicator for detecting the presence of glaucoma and the degree of glaucomatous optic neuropathy. Tremendous efforts including supervised learning^5–7^ and semi-supervised learning^8^ have been invested in automated segmentation of the optic disc and the optic cup, but the accuracy of computing CDR values remains a great challenge due to the large overlap and extremely weak contrast between the optic cup and the retinal limb region. Pathologically high IOP is a common symptom of glaucoma, but a proportion of glaucoma patients also have normal IOP, which is not specific enough to be a valid detection tool for a large number of glaucoma patients. RNFL thickness around the optic nerve head is another parameter more commonly used to diagnose glaucoma. Kozekanani et al.^9^ proposed a Markov boundary model to calculate the RNFL. But RNFL thickness may also be beyond the normal range in patients who suffer from other retinal pathologies and eye morphology (e.g., myopia). Visual field defect, measured and monitored by Kinetic tonometry, is a major symptom in patients with advanced glaucoma. Yousefi et al.^10^ proposed an expectation maximization (GEM) method to identify glaucomatous defect patterns. Ceccon et al.^11^ proposed the use of Bayesian networks for classification and clustering to explore early glaucoma and visual field testing. However, because measurement requires patient performance and attention, it leads to gaze deficits, false positives, false negatives, and other confounding errors^12^. The singlemodal methods lack other types of data comparisons for segmentation tasks and lack in-depth glaucoma subtype labels for classification tasks, which greatly limit their clinical application. Therefore multimodal methods have received more attention.

### Multimodal methods

In contrast, glaucoma clinicians deal with multimodal data from multiple sources when diagnosing, evaluating prognosis and deciding treatment plans. Multimodal diagnosis based on deep learning has become one of the challenges to improve the accuracy of glaucoma diagnosis. Hu et al.^13^ proposed a registered-fundus and multimodal vessel segmentation approach based on fundus photographs and OCT.However, this approach still suffers from issues such as artifacts. Shankaranarayana et al.^14^ constructed a fully convolutional network for optic disc cup segmentation using retinal images and ground truth depth images with OCT-based. Hervella et al.^15^ proposed a self-supervised pre-training method for the segmentation task using unlabeled multimodal image pairs consisting of retinography and fluorescein angiography (FA) images. However, FA is an invasive technique that requires the injection of a contrast agent. It has been replaced by more advanced non-invasive techniques, such as OCT. Current multimodal techniques still focus on using multiple medical images to compare with each other and improve segmentation accuracy.

## Results

In this section, the network skeleton of GMNN-Net is first changed to evaluate the performance under different backbones. Subsequently, ablation experiments are designed to demonstrate the effectiveness of introducing multimodality. Following that, it is discussed why and when it is valuable to introduce transfer learning from a pre-trained ImageNet CNN model. Finally, a visual comparison of different CAM methods and evaluation metrics are compared, and the Grad-CAM method is found to have the highest accuracy in adding interpretability to the model. GMNN-Net is implemented based on Keras and Pytorch. All experiments are performed on a tower workstation with an NVIDIA Tesla A100.

### Data preparation

**Table 1:** Example of a publicly available glaucoma datasets.

Dataset name	Quantity	Pixels	Feature	Generation distribution	Remarks
Drishti-GS^16^	101	2896 × 1944	OD/OC	40 ~ 80	Fundus photo
HRF^17^	15N/15G	3504 × 2336	Vessel segment	–	Fundus photo
ORIGA$^{-light}$^18^	650	3072 × 2048	OD/OC	40 ~ 80	Fundus photo
RIMONE^19^	118N/51G	–	OD/OC	–	Fundus photo
ACRIMA^20^	309N/396G	–	OD/OC	–	Fundus photo
OIA-ODIR	10000	–	–	–	Fundus photo
MESSIDOR^21^	1200	1440 × 960	DR		Fundus photo
LAG^22^	11760	1977 × 2594	–	53.6 (average)	Fundus photo
3D-OCTA^23^	316	640 × 400 × 400	vessel segment	$49.07\pm 17.56$	OCT &OCTA
INSPIRE-stereo	30	768 × 1019	OD/OC	–	3D Fundus+SD-CTA

N stands for normal, G stands for glaucoma, OD stands for optical disc, OC stands for optical cup.

**Table 2:** Comprehensive information of GM367.

(a) Data distribution					
Male	158 (43%)				
Female	209 (57%)				
Generation distribution	$40\sim 80$				
Average age	58.3				
Normal	44%				
POAG	24%				
PACG	12%				
SOAG	10%				
SACG	10%				

(b) Medical Image Information					
Category		No. of images	Category		No. of images
Left	Perimetry	365	Right	Perimetry	347
	OCT	356		OCT	324
	Fundus	360		Fundus	352
	RNFL	357		RNFL	341
	UBM	366		UBM	362
	OCTA	32		OCTA	31
CDR ≥ 0.65		289 + 244 + 257	CDR < 0.65		391 + 468 + 441
Follow ISNT rule		277 + 262 + 239	Not follow ISNT rule		403 + 450 + 459

Patients with suspected glaucoma can undergo a variety of tests in the clinic, such as measurement of IOP, ultrasound biomicroscopy (UBM) to see if the angle is open or closed, optical coherence tomography (OCT)and fundus images to view the retina and optic nerve. These tests focus on different diagnostic indicators of glaucoma and complement each other. The combination of these tests can be used to achieve the best clinical accuracy. Therefore, we cooperated with Shenyang Fourth People’s Hospital to construct a new glaucoma dataset (GM367), which includes 367 patients’ electronic medical records, 680 Heidelberg OCT, 712 color fundus photos , 698 RNFL thickness images,728 Ultrasound biomicroscopy(UBM) photo and some OCTA images and SLO. The electronic medical records contain human metadata such as age, gender, medical history, visual acuity, intraocular pressure, various specialty examinations and diagnoses. The dataset consists of the five most important glaucoma subclass labels, including normal (N), primary open-angle glaucoma (POAG), primary closed-angle glaucoma (PACG), secondary open-angle glaucoma(SOCG), and secondary closed-angle glaucoma (SACG). The Appendix Fig. 1 shows some typical samples of the five glaucoma subclasses, with more than 95% of all pathologies belonging to one of the five diagnostic classes. In practice, the task of clinicians is to distinguish between different glaucoma classes and make a specific diagnosis, therefore the construction of a glaucoma multiclass dataset is necessary.The detailed information is shown in the Table 2 and the Appendix Figs. 1 and 2.

### Data preprocessing

The purpose of image and metadata preprocessing is to reduce the effect of noise and imbalance classes in the datasetso as to increase the ability of models to learn important features hidden in metadata and images^24^.

The metadata includes two parts, one is the metadata such as gender, age, disease description, IOP, etc. in the electronic medical record of glaucoma patients, and the other part is the information extracted from the images, such as Cup and Disc Ratio (CDR), RNFL thickness, whether or not to follow ISNT rules, etc. The feature of the above data can be divided into numeric types and categorical types. Min-max normalization is used for numerical features, which scales and translates each feature into the interval [0,1]. The one-hot encoding method is applied for producing vectors and converting categorical features into dummy features,which can effectively prevent transformed categorical features from being assigned ordinal meaning^24^. IOP in glaucoma patients changes as the disease progresses, so the data are often recorded once a day and the missing values are processed with the mean insertion method for numerical values and the mode insertion method for categorical values.

The images in the dataset are collected by advanced Heidelberg OCT, 3D fundus camera, OCTA and other equipment, which can obtain data of higher quality and pixels. Although this allows to improve the accuracy of glaucoma diagnosis to some extent, it can significantly increase the training time of the model. Therefore, we adopted the method proposed by Xu et al.^25^ in which a bounding box of 1.5 times the radius of the optic disc is used to automatically crop around the optic disc. In their method, they use a basic CNN to find the most likely pixels in the optic disc region. Then, they classify these candidate pixels by using a threshold.

Data imbalance is a serious problem in classification tasks which severely affects classification accuracy. If the model is trained on imbalanced data, it usually classifies new samples as majority classes. From Table 4, we can find the imbalance of medical images among the five glaucoma classes. Appropriate data imbalance treatment method is necessary.Image enhancement is an effective processing method. We expand the number of images according to the ratio of each category, i.e., the fewer the number of images, the more the number of expansions. The method of data augmentation is to shift the images by 50 pixels in 8 directions (i.e. up, down, right, left, left, right, up left, right, down left and down right). Then, all images are flipped horizontally and rotated by 90, 180 and 270 degrees.

### Performance comparison of different CNN backbones for GMNNnet

The GMNN-Net consists of three branches: the first branch processes textual information from the patient’s electronic record which is composed of the convolutional, recurrent and transcriptional layers. The second branch is built on the M-Unet network for segmenting optic nerve vascular distribution features, calculating cup-to-disc ratios and extracting optic nerve fiber layer thicknesses, and then introducing image features into the metadata. The third branch focuses on glaucoma images and uses a series of state-of-the-art deep learning models as the backbone to capture the global and local details of glaucoma.

**Figure 1:**
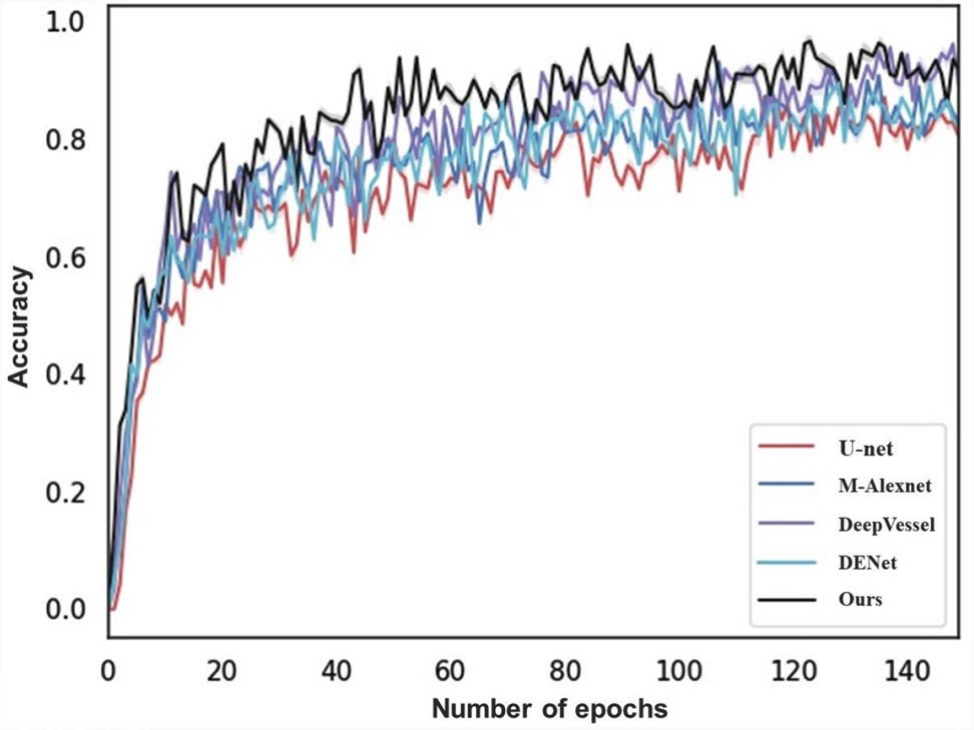
The ACC of different branch 2.

**Figure 2:**
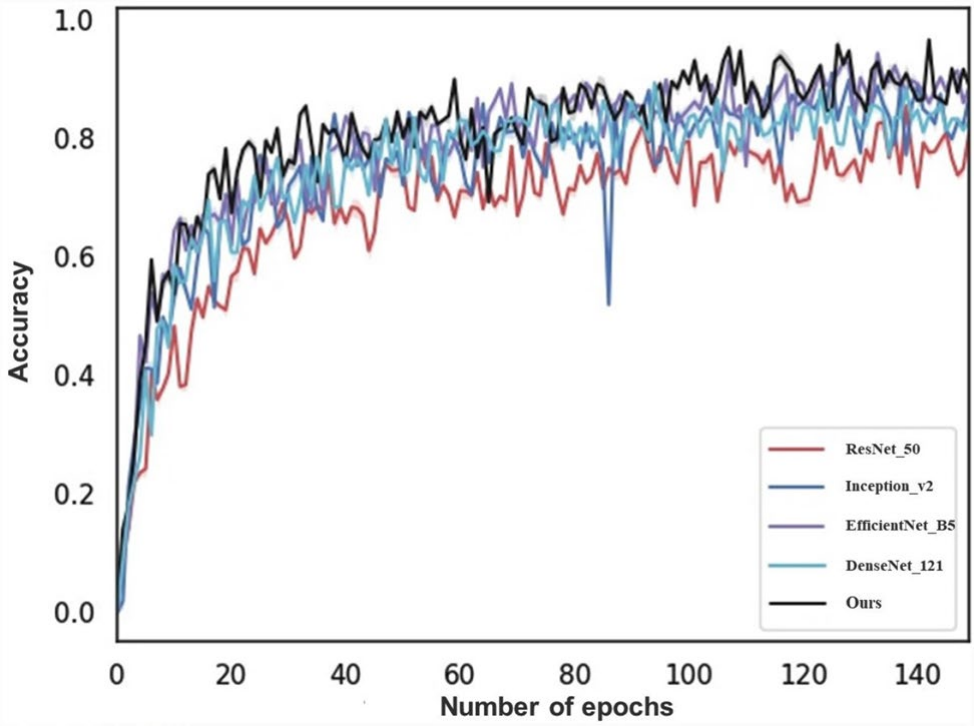
The ACC of different branch 3.

**Figure 3:**
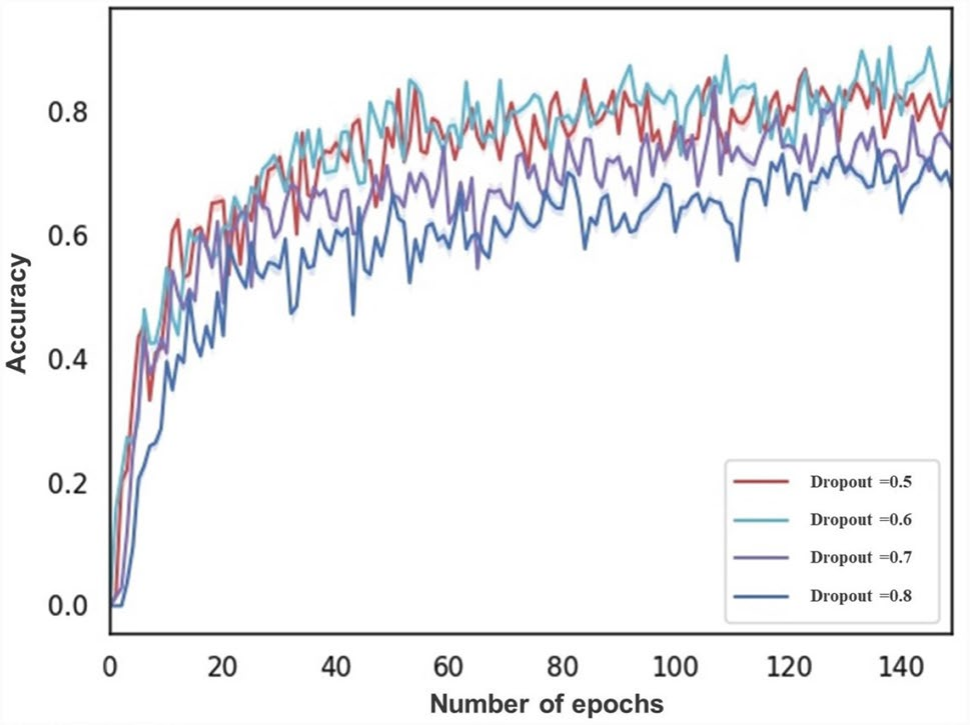
The ACC of different dropout.

**Table 3:** Results for each model and 5-fold cross validation.

Branch 1	Branch 2	Branch 3	Predict time	ACC	SPE	SEN	Fscore	AUC	95% confidence interval
$\checkmark$	U-net^26^	ResFormer	0.009	0.883	0.785	0.861	0.897	0.874	86.06–88.97%
	M-Alexnet^27^		0.010	0.896	0.817	0.878	0.902	0.885	86.76–89.91%
	DENet^28^		0.013	0.915	0.825	0.896	0.897	0.895	88.31–91.23%
	DeepVessel^29^		0.014	0.926	0.847	0.919	0.917	0.906	89.36–91.82%
	M-Unet	ResNet$\_$50	0.009	0.876	0.775	0.859	0.867	0.866	85.71–87.77%
		Inception$\_$v3	0.014	0.928	0.823	0.895	0.891	0.886	87.01–89.17%
		DenseNet$\_$121	0.010	0.896	0.798	0.843	0.867	0.879	86.32–88.92%
		Efficient$\_$B5	0.012	0.937	0.873	0.906	0.916	0.912	90.03–92.16%
Ours			0.012	0.951	0.886	0.932	0.943	0.939	93.35–94.25%

Two sets of comparison experiments are performed between GMNNnet and the well known CNNs. Since GMNNnet can access a very different set of input data (i.e. metadata) compared to previous studies, we fix the backbone of networks in branch 2 or 3 respectively and then embed the different neural networks in the other branch of the model for comparison. We compare the performance of segmentation networks such as Unet, M-Alexnet, DENet and DeepVessel with our proposed M-Unet in branch 2 (Fig. 1). Branch 3 compares the performance of convolutional networks such as ResNet, Inception, DenseNet and EfficientNet with our model (Fig. 2. This experiment is implemented based on the Kera and Pytorch frameworks, and all model weights are obtained by transfer learning. All are optimized using Adam with an initial learning rate of 0.0001, which is updated with the number of iterations. The batch size is equal to 64. The original patches are preprocessed and normalized to a single channel. For model evaluation, a 5-fold cross-validation is introduced. Thus after obtaining 5 values for area under the curve (AUC), accuracy, specificity, sensitivity and F-score, the mean and standard deviation of these values are calculated for each CNN architecture. Comparing the results in Table 3, it can be found that GMNNnet performs the best among all models. The significant improvement in SPE and AUC demonstrates the effectiveness of introducing multi-modality in GMNN-Net and making full use of the fundamental backbone. Simultaneously, the prediction time for individual patients is relatively fast, averaging less than 0.02 seconds, which is not significantly different from the fastest network. The fast prediction times suggest that our model can be used for routine clinical work. In addition, we fine-tune the parameter settings of Dropout and find that the probability of 0.6 works best, as shown in Fig. 3.

**Figure 4:**
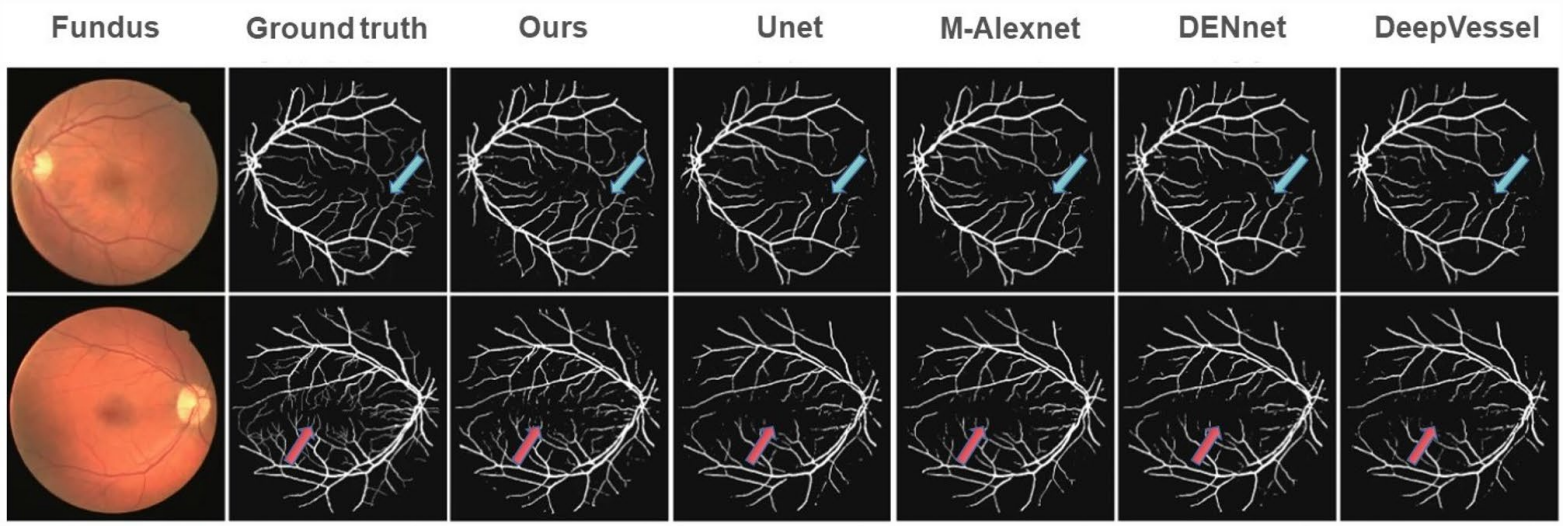
Visualization of retinal vessel segmentation based on fundus photos.

We selected segmented images of fundus photographs for visualization, and introduced the Dice metric for quantitative analysis. In the region indicated by the arrow in the Fig. 4, our model has more accurate segmentation accuracy for finer retinal vessels.The average Dice index is 0.98, which is 8% higher than M-Alexnet.

1 $$\begin{aligned} Dice(X,Y)=\frac{2\left| X \cap Y \right| }{\left( \left| X \right| + \left| Y \right| \right) }\Leftrightarrow Dice=\frac{2TP}{FP+2TP+FN} \end{aligned}$$

### Performance comparison of different modal

To demonstrate the effectiveness of introducing GMNNnet for glaucoma diagnosis,we performed two sets of ablation experiments, one to distinguish the role of each input modal in prediction accuracy and the other to demonstrate the necessity of inputting four medical images in branch 2 and branch 3. In Table 4, we evaluated these three branches to measure the contribution of each branch. The multimodal model outperforms any unimodal model in terms of mean ACC, SEN, SPE, and AUC, with the most significant improvement in sensitivity. The most significant improvement in sensitivity confirms the effectiveness of GMNN-Net. The output of the network showed an average improvement of 111%, 68% and 13% compared to branch 1, branch 2 and branch 3. Meanwhile, branch 3 contributed more to the accuracy of glaucoma diagnosis, which may be due to the fact that medical images can extract more effective features of glaucoma. Compared with branch 3, the multimodal output showed improvements of 3%, 13%, 3% and 8% in ACC, SEN, SPE and AUC, respectively.

**Table 4:** Comparison with different modal.

Branch 1	Branch 2	Branch 3	Modal	ACC	SEN	SPE	AUC
$\checkmark$	✕	✕	1	0.686	0.467	0.712	0.693
✕	$\checkmark$	✕		0.709	0.407	0.762	0.753
✕	✕	$\checkmark$		0.789	0.569	0.823	0.733
$\checkmark$	$\checkmark$	✕	2	0.820	0.512	0.858	0.717
$\checkmark$	✕	$\checkmark$		0.849	0.632	0.898	0.827
✕	$\checkmark$	$\checkmark$		0.922	0.847	0.915	0.921
$\checkmark$	$\checkmark$	$\checkmark$	3	0.951	0.865	0.932	0.962

### Comparison of random initialization training and transfer learning

CNN models in the multimodal neural network branch can either be learned from scratch or fine-tuned from a pre-trained model. Mainstream deep CNN architectures (e.g., ResNet, EfficientNet) contain tens of millions of free parameters to train and thus require a sufficiently large number of labeled medical images. On the other hand, collecting and annotating a large number of medical images still faces significant challenges. Numerous studies have demonstrated that transfer learning from ImageNet to other limited size datasets via CNN can learn deep models with better performance.

**Figure 5:**
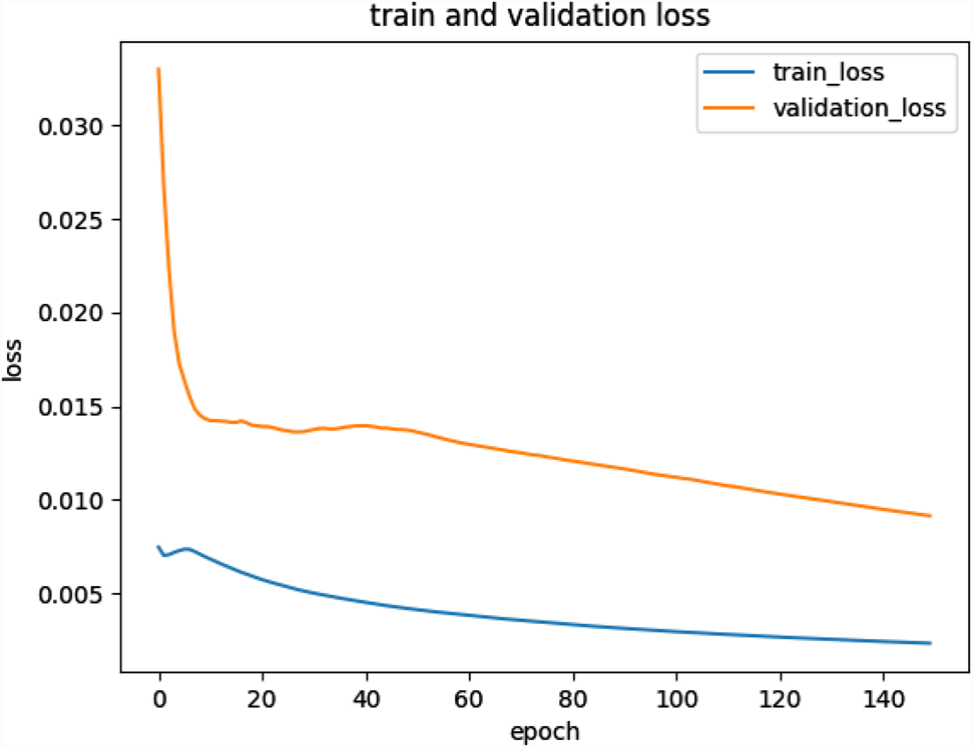
Model training using transfer learning method.

**Figure 6:**
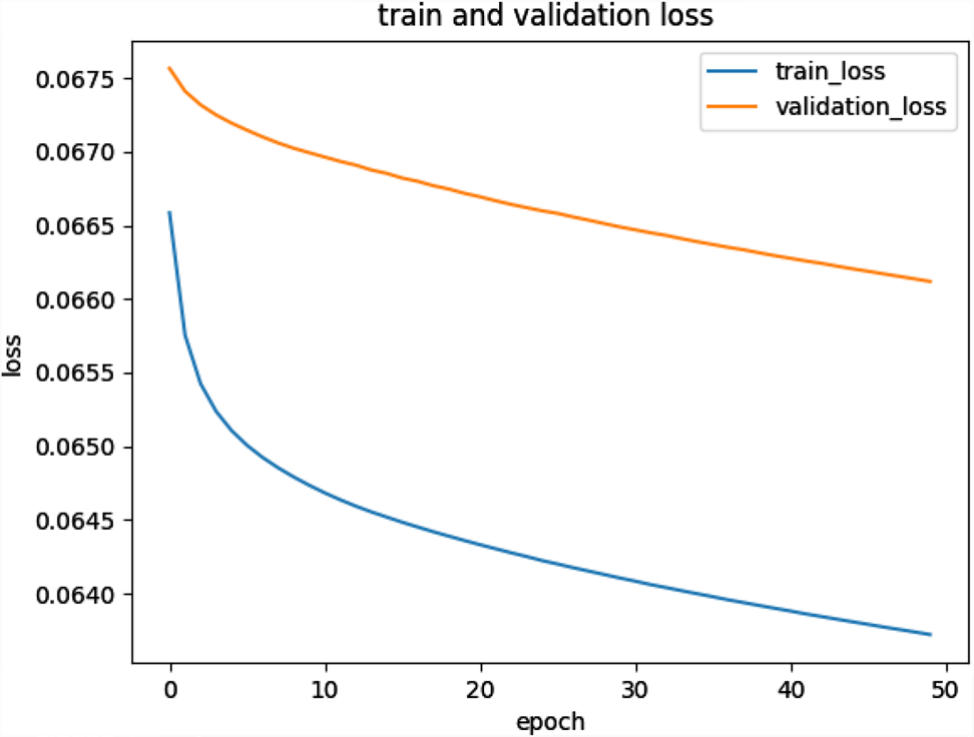
Model training using random initialization.

We conducted comparative experiments to determine whether we need to fine-tune the “end-to-end” CNN network to improve performance, rather than just training the final classification layer. For transfer learning, we followed the approach of^30^, where all CNN layers except the last layer are fine-tuned at a learning rate 10 times smaller than the default learning rate. The last fully connected layer was randomly initialized and trained on the glaucoma dataset to fit our classification task. Its learning rate is kept at the original 0.01. We also experimented with a CNN pre-trained on ImageNet and trained only the final classifier layer for the new glaucoma classification task. The parameters in the convolutional and fully connected layers are fixed and used as deep image extractors. After 20 epochs, the loss of the model with transferlearning is about 0.015 (Fig. 5), while the loss of the model trained from scratch is about 0.067 (Fig. 6). It is a good proof of the effectiveness of introducing the transferleaning method to solve the insufficient number of medical image datasets.

### Comparison of different CAM

**Figure 7:**
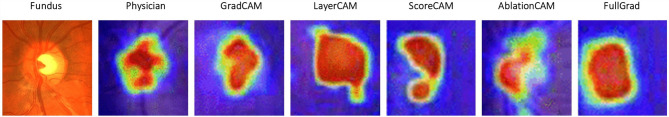
Attention map of fundus photos with different CAM methods.

Interpretability is very important in the medical field, which can explain which areas are the areas that clinicians pay more attention to in diagnosing glaucoma, and it is easier to build patients’ trust in intelligent systems and make them meaningfully integrated into daily life. We use the method proposed by Li et al.^22^. To mark the area that clinicians pay attention to, and design a comparative experiment to compare the accuracy of heat maps generated by different CAM methods. And Dice index is used for quantitative evaluation. Compared with many famous CAM methods, we found that the areas marked by GradCAM coincide more with the attention areas of doctors (Fig. 7), and the Dice index reaches 0.85 (Table 5). Although the recent work^31^ shows serious concern about its accuracy, especially in the limited data training, the model parameters are more accurate and there are no serious errors due to the transfer learning method used in the model.

**Table 5:** Comparison with different CAM using Dice.

Index	GradCAM	LayerCAM	ScoreCAM	AblationCAM	FullGrad
Dice	0.85	0.63	0.45	0.21	0.55

## Discussion

At present, the resolution of medical images often determines the accuracy of diagnosis. The resolution of medical images usually is over 1000, which is far beyond the $224 \times 224$ resolution of general image classification networks. If the images are fed directly into the network, the training time will be greatly increased. If the images are cropped, pathological features will be lost, which will lead to a decrease in diagnostic accuracy. How to increase the size of the perceptual field? This is crucial for object detection in high-resolution images, especially for medical diagnosis. Therefore, how to solve such a problem will soon be a question to be considered.

There are still several problems in the training process. First, the network parameters could not be optimized due to the large number of network parameters and the small medical dataset. When the HRF, a small medical image dataset, is used as the input to the neural network alone, the model cannot fully learn the relevant features of glaucoma. Second, the samples are unbalanced. For example, when we use the OIA dataset, due to the imbalance between glaucoma samples and normal samples, the fit is better in the training set but less accurate in the test set, leading to the overfitting problem. Therefore, we need to adjust the network according to the characteristics of the data.

## Conclusion

In this paper, we construct a new glaucoma dataset GM367, with five labels and multiple medical images. This is the first multimodal multiclassification dataset for glaucoma to our knowledge. Meanwhile, we construct a multimodal neural network GMNN-Net, which embeds a three-branch structure in the network and fuses textual and image information together at the end. A large number of experimental results show that the ACC, SEN, SPE and AUC of the multimodal glaucoma diagnosis model are improved by 1.4%, 1.3%, 2.6% and 2.7% respectively compared with the current deep learning method. The above work has three meanings for the clinical application of intelligent diagnosis of glaucoma. First, make the diagnostic label go deep into the glaucoma type, instead of judging whether it is glaucoma as in the current research. Second, the fusion of multimodal data greatly improves the accuracy of glaucoma diagnosis. Third, Grad-CAM method is added to increase the interpretability of the model, which is helpful to apply the model to clinical diagnosis and greatly alleviate the shortage of glaucoma professionals at present. Future work will be divided into two parts: we will further enrich the dataset and collect time-series data of IOP to transform the glaucoma diagnosis problem into a prediction problem and further improve the early detection of glaucoma. We will further improve the accuracy of the multimodal neural network and enhance the performance of the model.

## Methods

To make full use of the domain knowledge of glaucoma and to utilize multiple glaucoma medical images simultaneously, we propose a multimodal neural network GMNN-Net for multiclass diagnosis of glaucoma. The glaucoma multimodal neural network consists of three branches for processing basic metadata of patients, extracted features and glaucoma images. The three inputs are optional, allowing for data without a single model. The flowchart of GMNN-Net is shown in figure. The first branch processes textual information from the patient’s electronic records, processed by convolutional, recurrent and transcriptional layers, with the aim of obtaining a feature matrix of glaucoma disease keywords. The second branch was built based on the U-Net network for extracting optic nerve vascular distribution features from Heidelberg OCT, calculating cup-to-disc ratios from fundus photographs and eye angle opening from UBM, and analyzing the thickness of the RNFL. Domain-specific knowledge was added to catboost through the first two branches. The third branch focuses on glaucoma images, using a series of state-of-the-art deep learning models as a backbone to capture global and local details of Heidelberg OCT images and optic nerve fiber layer thickness images. We apply the gradient-weighted class activation mapping $\left( Grad-CAM\right)$ to construct interpretable modules, which allows us to achieve high accuracy and good interpretability.

**Figure 8:**
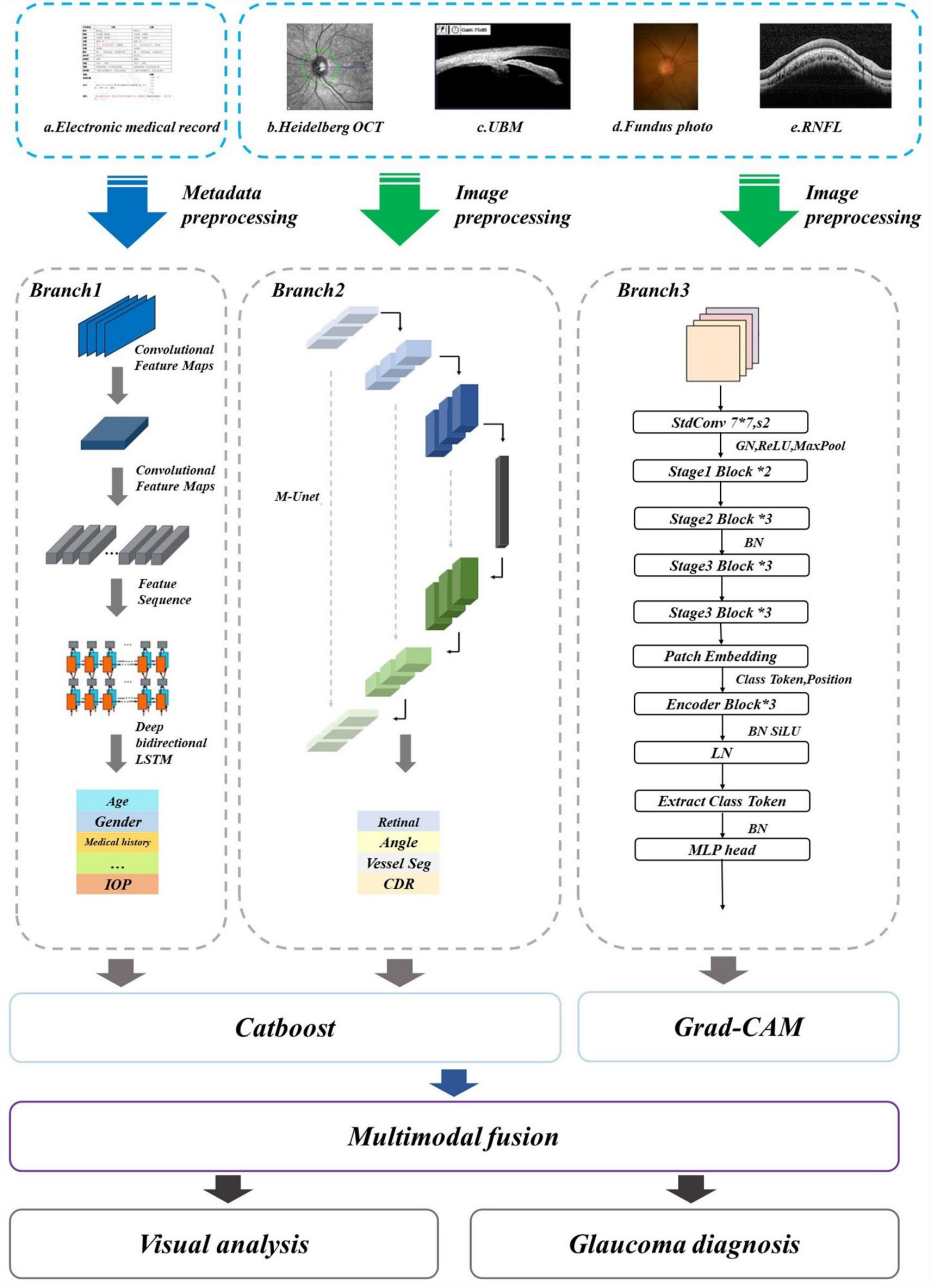
Flowchart of GMNNnet.

### The details of branches in glaucoma multimodal neural networks

#### Branch 1

In the multimodal glaucoma database, electronic medical records of 367 patients have been collected. These will be used to extract keywords for glaucoma disease as metadata input to the first branch of the glaucoma multimodal neural network to assist the diagnosis of glaucoma. The first branch consists of a convolutional layer, a recurrent layer and a transcription layer, as shown in Fig. 8.

The convolutional layer consists of the convolutional layer and the maximum pooling layer, and the fully connected layer is removed. It is used to extract glaucoma keywords from the input image and embed them into a high-dimensional space, so that the semantic relationships between words are better represented. The use of vectors avoids the “dimensional disaster” problem of word representation. Each feature vector in the feature sequence is generated from a feature map.

The recurrent layer is a bi-directional RNN that predicts each sequence of features generated in the convolutional layer as a label distribution. The first reason for choosing RNN is its strong ability to capture the contextual information of the sequence. In the above feature extraction, a wide character may have several consecutive sense field descriptions. Using the context for image-based sequence recognition is more effective than processing a character alone. And for some ambiguous characters, they will be well distinguished after observing their contextual information. The second reason is that RNN can also back propagate for weight update, so CNN and RNN can be connected into a complete network. The third reason is that RNNs can process sequences of any length, so in this case, images of any width can be processed.

The role of the transcription layer is to convert the predictions generated by the RNN into labeled sequences. The conditional probability method defined by the connectionist temporal classification layer is used to obtain the probability of the label sequence conditional on the prediction $y=y_{1},\dots ,y_{t}$ generated by the RNN. Therefore, the negative log-likelihood of this probability can be used as the objective function for training the network.

2 $$\begin{aligned} p\left( l|y \right) = \sum _{\pi : B\left( \pi \right) =1 }p\left( \pi |y \right) =\sum _{\pi : B\left( \pi \right) =1}\prod _{t=1}^{T}y_{\pi _{t} }^{t} \end{aligned}$$

Assume that the output after the current layer is $y=y_{1},\dots ,y_{t}$,every $y_{t}\in {\mathbb {R}}^{L^{'}}$ is the probability distribution over the set *L*, *L* contains all labels in the task and a blank label, a sequence-to-sequence function mapping B is defined on the sequence $\pi \in L^{'T}$ , $y_{\pi _{t} }^{t}$ is the probability that there is a label $\pi _{t}$ at time t.

#### Branch 2

**Figure 9:**
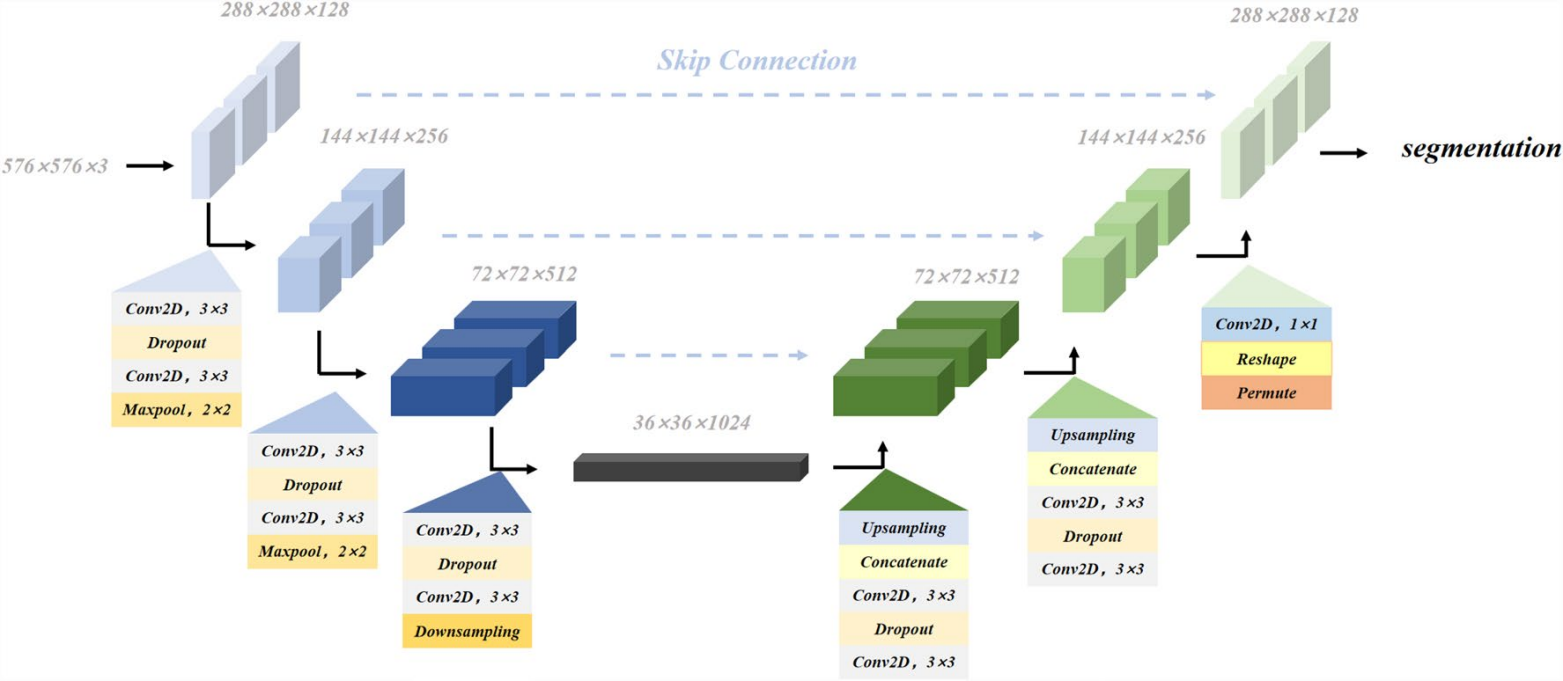
Flowchart of M-Unet.

The goal of the second branch is to design our M-Unet architecture (shown in the Fig. 9) based on the well-known U-net model. The network processes four glaucoma medical images simultaneously, including Heidelberg OCT,fundus photographs, UBM and RNFL images corresponding to extracted retinal lesions, optic nerve vessels, angle openings and CDR. Why do we choose different medical images to extract the corresponding features? OCT is a non-invasive optical imaging modality that uses coherent light to capture 3D structural data of the retina at micron resolution. Compared to color fundus imaging techniques, OCT allows to obtain more detailed information about the retinal structure, and thus we choose OCT to extract glaucomatous retinal lesions (such as narrowing along the optic disc, deepening of the optic cup, nasal displacement of the retinal vessels, choroidal atrophy or defects in the optic fiber layer). Widely used quantitative retinal vascular metrics (e.g., vessel density and vessel tortuosity) are important indicators for diagnosing diseases such as glaucoma, hypertension, and diabetic retinopathy. Most RV segmentation tasks are performed on color fundus images. Due to the limited ability of OCT images to present blood information, only a few methods have been reported. Therefore, we used fundus photographs to extract quantitative indicators of retinal vessels. UBM is an instrument for atrial angle examination that can visualize the atrial angle structure more clearly compared to atrial angle microscopy, and we chose UBM to extract the angle opening data. In addition, the RNFL image is an unfolded image of the retina, and calculating the CDR by measuring the RNFL thickness can be more accurate than dividing the optic cup and optic disc by fundus photographs.Branch 1 and Branch 2 are mixed together as metadata and processed with Catboost classifier.

#### Branch 3

Branch 3 combines ResNet with Transformer. ResNet made some adjustments, first using StdConv2d instead of traditional Conv2d for the convolution layer, and then replacing all BatchNorm layers with GroupNorm layers. Stages 1, 2, 3, and 4 are stacked 3, 4, 6, and 3 times respectively in the original Resnet 50 network. But in this network, they are 2, 3, 3, and 2, respectively. After feature extraction through ResNet50 Backbone, the obtained feature matrix shape is [14, 14, 1024], and then input it into Patch Embedding layer. Note that the kernel size and stride of convolution layer Conv2d in Patch Embedding are changed to 1, which is only used to adjust channel. Note that you need to add [class] token and Position Embedding before typing Transformer Encoder. Then, the Encoder Block is stacked three times, which consists of Layer Norm, Multi-Head Attention, and Dropout/DropPath. Finally, we only need to extract the corresponding results generated by [class] token, and then get the final classification results through MLP Head. It should be noted that when training in ImageNet, it is composed of Linear + tanh activation function + Linear. However, when migrating to glaucoma data, only one Linear is needed.

### Interpretable vision module based on grad-CAM

It is very difficult for doctors to use their eyes to directly identify some tiny features of glaucoma. Interpretable vision module support doctor in their effort to distinguish the differences between similar features of glaucoma, highlight the key areas of concern in glaucoma images, and help promote classification results.

For images, abnormal regions are highlighted in the form of visual heat maps. The region of interest (ROI) shows the high clinical relevance of glaucoma lesions. Grad-CAM$^{++}$ uses the global average of the gradients to calculate the weights.

3 $$\begin{aligned} Y^{c} =\sum _{k} w_{k}^{c} \cdot \sum _{i}\sum _{j}A_{i,j}^{k} \end{aligned}$$

where $Y^{c}$ means that the score of a certain class is the dot product of weight $w_{k}^{c}$ and feature map $A_{i,j}^{k}$.

The Grad-CAM$^{+}$ heat-map is a weighted combination of feature maps and can be expressed as follows:

4 $$\begin{aligned} L_{i,j}^{c} =\sum _{k}w_{k}^{c} \cdot A_{i,j}^{k} \end{aligned}$$

and $w_{k}^{c}$ can be calculated as follows

5 $$\begin{aligned} w_{k}^{c}=\sum _{i}\sum _{j}\alpha _{ij}^{kc}\cdot relu\left( \frac{\partial Y^{c} }{\partial A_{ij}^{k} } \right) \end{aligned}$$

where $\alpha _{ij}^{kc}$ is the weight coefficient for the pixelwise gradients for class c and convolutional feature map $A_{ij}^{k}$.

### Multimodality fusion

Imbalanced data are common in the medical field, which makes the classifier focus more on the major classes but neglect the minor classes. It results in a low sensitivity to the minor classes and a low specificity to the major classes,which can be addressed to a certain degree by revising the loss function.

The categorical cross-entropy loss is a popular loss function in multiclass classification learning. It assigns the same weight to each class, which leads to little attention to the minor classes and results in a low sensitivity for underrepresented classes. To overcome the effect of imbalanced data, we introduces focal loss as the loss function. Focal loss is a variant of the categorical cross-entropy loss, which has been proposed for handling imbalance data.

6 $$\begin{aligned} Focal_Loss=-\sum _{i=0}^{N} \sum _{c=0}^{C} \alpha _{i,c} y_{i,c}\left( 1-p_{i,c} \right) ^{\gamma }\log {p_{i,c}} \end{aligned}$$

The model based on the multimodal glaucoma dataset can diagnose different disease types of glaucoma, which is more in line with the clinical diagnosis. The accuracy of the diagnosis can be improved by using three branches to process the multimodal data and the mutual validation between the data. The introduction of Grad-CAM adds interpretability to the model and makes it easier to build patients’ trust in the intelligent system. The introduction of transfer learning solves the problem of insufficient number of medical imaging datasets.

## Supplementary Information

Supplementary Information.

## Data Availability

The datasets generated during and analysed during the current study are available from the corresponding author on reasonable request. We will consider making the datasets available to the public when the study is completed.
