# Supplementary material for: A transfer learning-based multimodal neural network combining metadata and multiple medical images for glaucoma type diagnosis
Source: Sci Rep. 2023 Jul 26;13:12076. doi: 10.1038/s41598-022-27045-6 (PMC10372152; doi:10.1038/s41598-022-27045-6)
Supplement: Supplementary file 1 — Supplementary Information. [file 41598_2022_27045_MOESM1_ESM.pdf]

Appendix

Dataset image

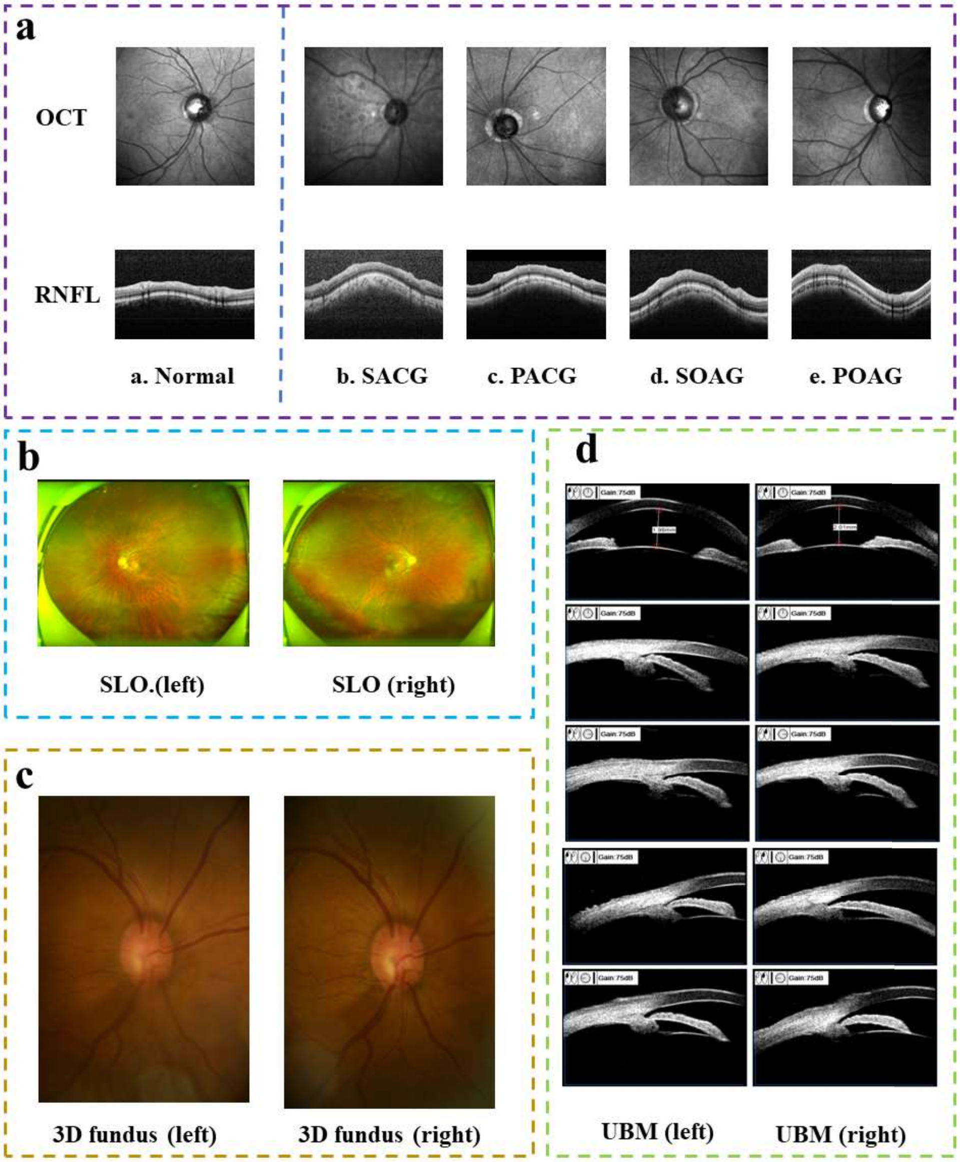

Figure 1. **a.**Heidelberg OCT adopts Eye-Tracking real-time eye tracking technology and ART real-time superposition noise reduction technology. **b.**SLO is a non-invasive examination, which observes Three-dimensional imaging of fundus blood vessels and helps ophthalmologists to detect subtle lesions in the eye at an early stage. **c.**Fundus images are widely used for [redacted] anterior segment tissue structure by high frequency ultrasound technology.

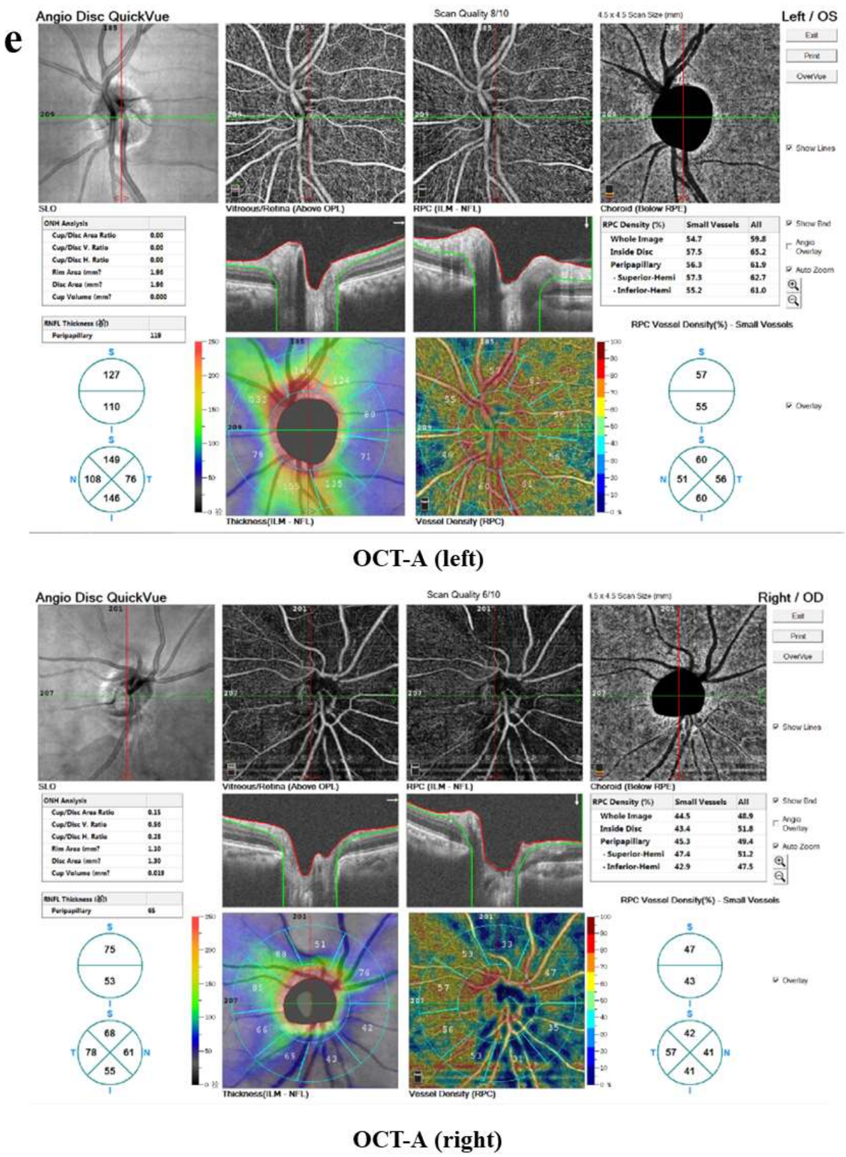

Figure 2. **e.**OCTA is a new non-invasive fundus imaging technique, which can identify retinal and choroidal blood flow information with high resolution and image retinal and choroidal microvascular circulation in vivo.

Glaucoma features

Cup-to-Disc Ratio

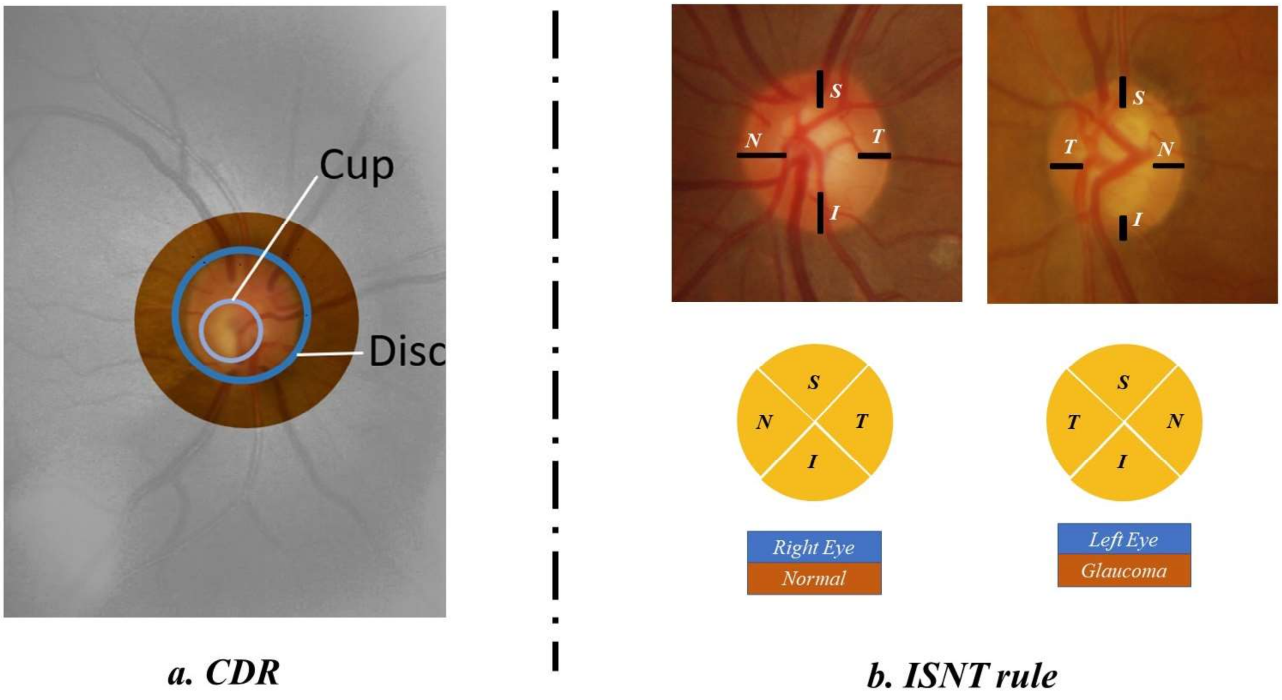

Figure 3. a. The figure shows the optic disc and optic cup regions, with the optic cup in the gray circle and the optic disc circled in blue. b. Inferior Superior Nasal Temporal (ISNT) rule.

In clinical diagnosis, CDR is an important feature for doctors to judge whether patients suffer from glaucoma. The CDR is calculated by measuring the proportion among the size of the optic nerve $(disc)$ and the size of the excavation inside the optic nerve produced by the increasing eye's pressure (cup) . As shown in the figure3(a).

$$HCDR = \frac{H_{cup}}{H_{disc}} \tag{7}$$

$$VCDR = \frac{V_{cup}}{V_{disc}} \tag{8}$$

$$ACDR = \frac{A_{cup}}{A_{disc}} \tag{9}$$

where HCDR is the horizontal cup-to-disc ratio, VCDR is the vertical cup-to-disc ratio, and ACDR is the regional cup-to-disc ratio.

ISNT rule

[redacted]

the Superior. The Nasal is narrower than the Inferior or Superior, and Temporal is the narrowest. As shown in the figure3(b)

$$Inferior > Superior > Nasal > Temporal \tag{10}$$

Disc Damage Likelihood Scale

$(DDLS)$ is a scale for calculating the probability of optic disc injury, that is, the severity of the disease.

$$DDLS = \frac{MinRIM_{width}}{DD} \tag{11}$$

where MinRIM width is the minimum width of the RIM, and DD is the disc diameter.

Glaucoma Risk Index
(GRI) is a combination of major components

$$GRI = 6.8375 - 1.1325\,(PC1) + 1.6500\,(PC2) + 2.7225\,(PC3) + 0.6750\,(PC4) + 0.6650\,(PC5) \tag{12}$$

[redacted]

If the range of GRI is (8.68 ± 1.67), the eye is considered normal, and if the range is (4.84 ± 2.08), the eye is considered abnormal.

Hyperparameters setting

The dataset was divided into a 75% training set and a 25% test set with a hierarchical training-test segmentation method to [redacted] experiments are implemented based on the Kera and Pytorch frameworks, and all models are fine-tuned for 50 epochs using Adam optimization with an initial learning rate of 0.0001,which is updated with the number of iterations. The batch size is equal to 16. Dropout is set to 0.6 to relieve overfitting.
